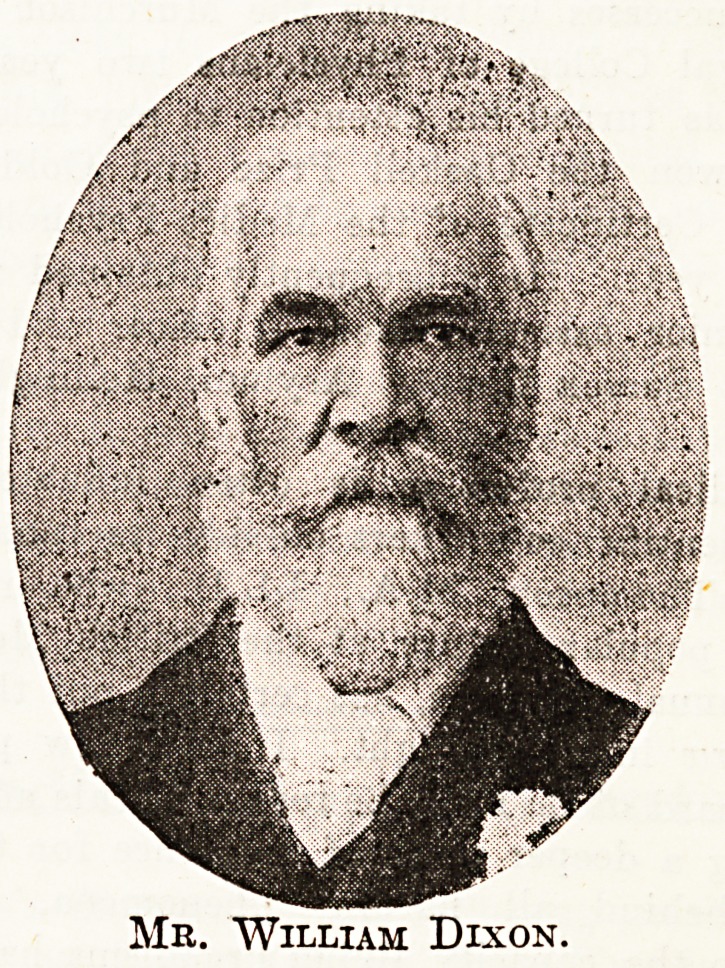# A Newcastle Hospital Pioneer

**Published:** 1914-08-15

**Authors:** 


### A Newcastle Hospital Pioneer.

MR. WILLIAM DIXON.

There has just retired from the staff at the Elswick Works of Sir W. G. Armstrong, Whitworth and Company, Limited, Newcastle-upon-Tyne, Mr. William Dixon, whose services to local medical charities are worthy of special note. Mr. Dixon has for over forty-two years been employed as workman and foreman in the great Elswick Works, where he has enjoyed the confidence of his employers and on many occasions been in consultation with the principals, including the late Lord Armstrong, the late Mr. W. D. Cruddas, Sir Andrew Noble, Bart., and others.

Mr. Dixon's special work on behalf of charities began in 1886 when, in response to an appeal issued by the then Chairman of the Newcastle Royal Infirmary, Mr. N. G. Clayton, there was commenced on Mr. Dixon's initiation the "Elswick Works Medical Charities Fund." Mr. Dixon organised and presided over a series of shop meetings at the Works. The committee finally adopted a scheme embodied in the above name which provided for weekly contributions deducted at the pay office. During the years 1886 to 1913 this fund, under Mr. Dixon's chairmanship, has distributed to medical charities over £63,000, of which the Newcastle Infirmary has received nearly £39,000.

Mr. Dixon was also chairman of a committee which promoted and conducted the scheme embodied in the Elswick Works Accident Compensation Fund which existed from 1881 to 1897. This fund was contributed to by both workmen and employers. Its total receipts amounted to £62,121. The distribution of benefits under this fund was of great service to injured workmen prior to the arrangement made under the Workmen's Compensation Acts which terminated the fund in 1897. The two funds having for their object the relief of human suffering have produced a grand total of over £125,000. Mr. Dixon will be remembered as a pioneer in the organisation of workmen's contributions, and, though he has retired from the services of Armstrong, Whitworth and

Mr. William Dixon.

Company, he is still chairman of the Medical Charities Fund.

He has, however, for over thirty years been a life governor of the Royal Victoria Infirmary and is also a member of the selection committee of that institution. He also represents the workmen on the governing body of several other charities. Mr. Dixon is a well-known figure in the life of Newcastle and district, and has served the cause of religion and charity as a preacher and platform advocate for over forty years.

## Figures and Tables

**Figure f1:**